# 780 nm Narrow Linewidth External Cavity Diode Laser for Quantum Sensing

**DOI:** 10.3390/s24227237

**Published:** 2024-11-13

**Authors:** Junzhu Ye, Chenggang Guan, Puchu Lv, Weiqi Wang, Xuan Chen, Ziyi Wang, Yifan Xiao, Linfeng Zhan, Jiaoli Gong, Yucheng Yao

**Affiliations:** 1 School of Science, Hubei University of Technology, Wuhan 430068, China; wahahaha@oeslab.com.cn (J.Y.); weiwiki@oeslab.com.cn (W.W.); fan@oeslab.com.cn (Y.X.); huabandiyishenqing@oeslab.com.cn (L.Z.); gongjl@hbut.edu.cn (J.G.); 2 Laboratory of Optoelectronics and Sensor (OES Lab), School of Science, Hubei University of Technology, Wuhan 430068, China; lv@oeslab.com.cn (P.L.); ersan@oeslab.com.cn (X.C.)

**Keywords:** external cavity diode laser, narrow linewidth, tunable range, interference filter

## Abstract

To meet the demands of laser communication, quantum precision measurement, cold atom technology, and other fields for narrow linewidth and low-noise light sources, an external cavity diode laser (ECDL) operating in the wavelength range around 780 nm was set up with a Fabry–Pérot etalon (F–P) and an interference filter (IF) in the experiment. The interference filter type ECDL (IF–ECDL) with butterfly-style packaging configuration has continuous wavelength tuning within a specified range through precise temperature and current control and has excellent single-mode characteristics. Experimental results indicate that the output power of the IF–ECDL is 14 mW, with a side-mode suppression ratio (SMSR) of 54 dB, a temperature-controlled mode-hop-free tuning range of 527 GHz (1.068 nm), and an output linewidth of 570 Hz. Compared to traditional lasers operating at 780 nm, the IF–ECDL exhibits narrower linewidth, lower noise, and higher spectral purity, and its dimensions are merely 25 × 15 × 8.5 mm^3^ weighing only 19.8 g, showcasing remarkable miniaturization and lightweight advantages over similar products in current research fields.

## 1. Introduction

Narrow linewidth lasers (NLLs) possess excellent temporal coherence and are widely used for coherent optical communication [1], quantum precision measurement [2,3,4], spectral analysis [5,6], and life sciences. The superior output characteristics of NLLs confer exceptionally high spectral purity, outstanding spectral stability, and minimal susceptibility to external interference, thereby permitting the provision of a stable output signal. Historically, the field of atomic, molecular, and optical (AMO) physics has been built upon experiments with NLLs, allowing unprecedented control over atoms and molecules [7]. The characteristics of laser linewidth have emerged as a pivotal factor in advancing the development of these scientific fields. At present, commercially available narrow linewidth lasers generally have linewidths below 100 kHz. Achieving narrower linewidth laser outputs and mass production has become a hot research field.

The linewidth theory was first proposed by Schawlow and Townes [8]. Twenty years later, Lang and Kobayashi [9] applied external cavity optical feedback technology to diode lasers and suggested that increasing the external cavity structure could narrow the laser linewidth, allowing for wavelength tuning by adjusting the external cavity length. H. Laurent’s team further pointed out that the reduction of the linewidth in external cavity lasers is proportional to the feedback level. The optical feedback technology takes advantage of the inherent high-frequency sensitivity of diode lasers to injected light, effectively reducing laser noise [10]. This technique can effectively suppress phase noise, thereby reducing the laser linewidth [11]. Xiang Huang proposed that increasing the external cavity can change the quality factor (Q) of the resonator by affecting the photon lifetime, thereby influencing the linewidth [12]. Building on this foundation, scholars successfully narrowed the laser linewidth to 1.5 MHz later by using a diffraction grating to construct the external cavity structure [13]. After 1988, ECDLs utilizing a narrow-bandwidth IF and a semitransparent mirror for external feedback were developed, enhancing their stability against external perturbations [14,15,16]. Due to the interference filter having only a single transmission peak, some researchers have used a single IF structure to achieve narrow linewidth output at different wavelengths for various applications. The output linewidth commonly ranges from 20 kHz to 100 kHz at both 780 nm and 852 nm [17,18,19,20,21]. In recent years, focusing on improving the practicability and environmental adaptability of lasers, scholars have conducted new explorations based on the traditional external cavity structure with a single IF. The research performed by Martin demonstrated that multiple frequency-selective devices instead of an IF were able to significantly reduce the spectral linewidth of lasers [22]. This discovery has opened up new avenues for improving laser performance. Notably, in 2019, Rui Chang’s team demonstrated that the transmission bandwidth of a dual-IF combination is smaller than that of a single IF. Using the dual-filter structure, they successfully achieved a narrow linewidth laser output of 96 kHz at a wavelength of 852 nm. In contrast, the linewidth of the laser output using a single filter structure was 176 kHz. The advantages of multi-frequency selection device structures were highlighted [23]. Additionally, some researchers have found that the combination of F–P and IF as frequency-selective elements can achieve even narrower linewidths. Since the F–P has multiple transmission peaks, its free spectral range can transform the gain spectrum of the diode laser into a frequency comb, which can then be further narrowed through secondary filtering with the IF. Ning Wang and Lv’s team utilized this combination of external cavity frequency-selective elements to achieve ultra-narrow linewidth laser outputs of 7 kHz and 3 kHz at a wavelength of 1550 nm, further demonstrating the significant potential of this composite frequency-selective structure in enhancing laser performance [24,25]. Given the above research achievements, we are working on applying this frequency selection structure, which combines an F–P and an IF, to the 780 nm laser. Our aim is to achieve a similarly excellent narrow linewidth laser output at this wavelength and fill the research gap in this field.

In this work, we present a miniaturized narrow linewidth IF–ECDL packaged in a butterfly-style package. Operating at 780 nm, the IF–ECDL distinguishes itself by its relatively small footprint and superior output performance. By utilizing a passive external cavity and partial mirror feedback, the linewidth is substantially narrowed, while the combination of an F–P and an IF enables precise control of laser frequency and single-mode output, making it ideal for high-precision light source applications, including quantum precision measurements.

## 2. Theoretical Analysis

### 2.1. Linewidth Analysis of External Cavity Laser

#### 2.1.1. Natural Linewidth

The linewidth of a laser is predominantly influenced by phase noise, and it can be derived from the phase noise of the laser itself. Experimental investigations have revealed that the linewidth value $\Delta v_{0}$ of diode lasers exceeds the prediction made by the Schawlow–Townes formula. To account for this discrepancy, an additional linewidth enhancement factor α was introduced, which allows for a more accurate expression of the natural linewidth of the laser, as given by [26]

(1) $$\Delta v=\left(1+\alpha^{2}\right)\frac{v_{g}^{2}hvgn_{sp}\alpha_{m}}{8\pi P_{0}}$$

**Figure 1:**
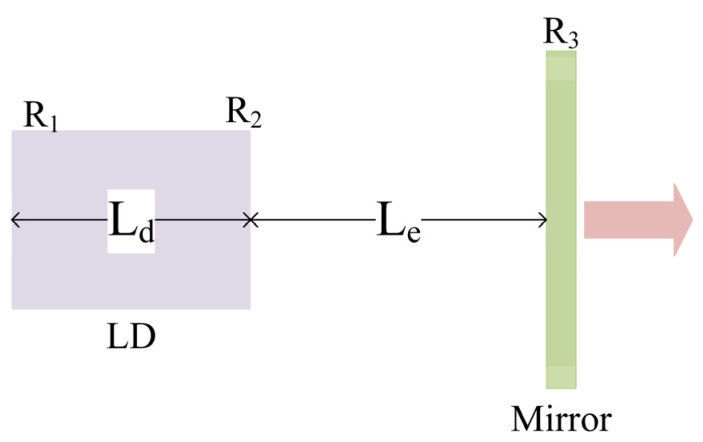
Basic model of the ECDL.

Here the linewidth enhancement factor α is influenced by the material properties of the diode laser and the injection current, and it typically ranges from 2 to 6. $v_{g}$ is the group velocity, and $n_{sp}$ is the spontaneous emission factor, being approximately 2 for diode lasers; hv is the photon energy; g is the chip gain; and $a_{m}$ is the output loss. When the laser works in a steady state, $g=a_{in}+a_{m}$, where $a_{in}$ is the internal loss. The simplest external cavity laser structure is shown in Figure 1, consisting of a diode laser and a plane mirror. The internal cavity length is denoted as $L_{d}$, and the external cavity length is denoted as $L_{e}$. The reflectivities of the left and right end faces are represented as $R_{1}$ and $R_{2}$, respectively, while the reflectivity of the mirror is $R_{3}$. The output loss of the diode laser can be expressed as:

(2) $$a_{m}=\frac{1}{2L_{d}}\ln\left(\frac{1}{R_{1}R_{2}}\right)$$

The laser output power, denoted as $P_{0}$, can be expressed in terms of the injection current into the laser, as given by

(3) $$P_{0}=\frac{a_{m}hv\left(I-I_{th}\right)}{gq_{e}}$$

where *I* is the injection current, $I_{th}$ is the threshold current, and $q_{e}$ is the electron charge. Using Equations (1)–(3), the natural linewidth of the diode laser can be further derived as:

(4) $$\Delta v=\frac{q_{e}v_{g}^{2}g^{2}n_{sp}\left(1+\alpha^{2}\right)}{8\pi \left(I-I_{th}\right)}$$

It can be seen that for a specific type of diode laser, parameters such as its intrinsic cavity length and facet reflectivities, as well as the injection current, have a significant impact on the natural linewidth. Increasing the current, which results in an increase an output power, is beneficial for reducing the linewidth.

#### 2.1.2. Reduce the Outer Cavity Linewidth

ECDLs can effectively reduce the linewidth, which mainly manifests in two ways: firstly, incorporating an external cavity is equivalent to increasing the effective cavity length of the laser, which leads to a narrower linewidth; secondly, the introduction of external cavity feedback enhances stimulating radiation, suppresses spontaneous radiation, and further narrows the linewidth of the output laser. The linewidth of the ECDL can be given as [27,28]:

(5) $$\Delta v_{0}=\Delta v\frac{1}{{\left(1+\tau_{e}/\tau_{d}\right)}^{2}}$$

$\tau_{d}$ and $\tau_{e}$ are the round-trip times of photons within these cavities. From Equation (5), it is evident that the linewidth $\Delta v_{0}$ of an ECDL is related to the external cavity length $L_{e}$. Consequently, extending $L_{e}$ effectively reduces the linewidth. Generally, the external cavity length in a diode laser significantly exceeds its intrinsic cavity length. Theoretically, this makes a reduction in linewidth by several orders of magnitude, even below 10 kHz.

### 2.2. Frequency Selection Principle

For an ECDL, the longitudinal mode spacing is given by Equation (6), where $L_{d}$ is the lengths of the internal cavity length, and $n_{d}$ is the refractive index of the active region.

(6) $$\Delta \lambda_{e}=\frac{\lambda^{2}}{2\left(L_{e}+n_{d}L_{d}\right)}$$

As indicated by Equation (6), the spacing of the longitudinal modes is inversely proportional to the length of the external cavity. Because the length of the external cavity is significantly bigger than the intrinsic cavity length, the spacing between the laser’s resonant modes decreases, resulting in a reduced frequency difference between adjacent modes. Through proper cavity design and gain control, the laser more easily achieves tuning by selectively amplifying one mode; however, at the same time, the requirement for frequency selection increases. In this study, a highly precise F–P combined with an IF was utilized to achieve frequency selection for the laser. The variation in the transmission rate of the F–P for different wavelengths is given by [21]

(7) $$T=\frac{1}{1+\frac{4R}{{\left(1-R\right)}^{2}}\sin^{2}(\frac{2\pi n_{FP}d\cos\theta }{\lambda })}$$

where R, $n_{FP}$, and d are the reflectivity, refractive index, and thickness of the F–P, respectively, and θ is the angle of incidence of the light. According to Equation (7), the reflectivity of the F–P should be appropriately increased to achieve high-finesse frequency selection. However, since a single F–P has multiple transmission peaks, it can easily result in the laser operating in a multi-longitudinal mode oscillation. By utilizing an appropriate design of the IF, single-mode frequency selection can be achieved.

## 3. Experiment and Result Analysis

### 3.1. Design and Manufacturing Process of IF–ECDL

**Figure 2:**
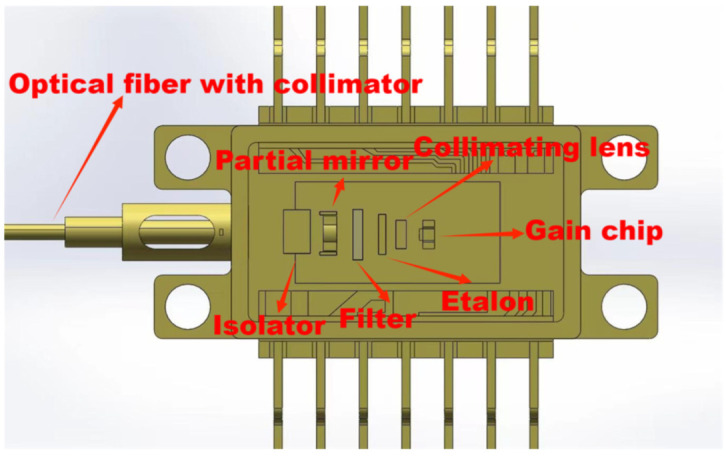
Structure diagram of butterfly IF–ECDL.

The internal structural diagram of the IF–ECDL was shown in Figure 2, which indicated the integration of a gain chip, collimating lens, F–P, IF, partial reflecting mirror, optical isolator, fiber collimator, and polarization-maintaining fiber. This configuration optimized the external feedback mechanism to mitigate the influence of intracavity modes. To finely select a single wavelength, the light emitting from the gain chip is collimated and then passes through the combination of the F–P etalon and IF. The light beam oscillates and amplifies within the resonant cavity formed by the partial reflector and the rear surface of the gain chip, ultimately achieving a single-frequency laser output once the threshold condition is reached. Finally, the laser was efficiently coupled into the polarization-maintaining fiber through the fiber collimator, enabling stable transmission.

**Figure 3:**
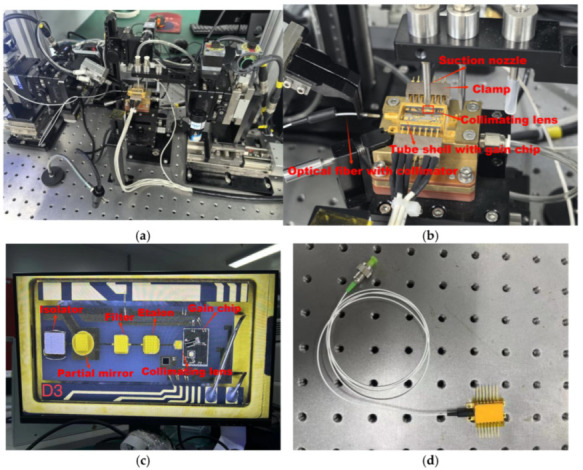
Manufacturing of the IF–ECDL with the automatic coupling device. (**a**) Installing the collimating lens with the automatic coupling device; (**b**) Partial enlargement of the coupling device; (**c**) Internal structure of the IF–ECDL; (**d**) Butterfly-shaped IF–ECDL.

An automated coupling device that integrates machine vision with automatic control technology was used to manufacture the IF–ECDL. The specific manufacturing procedure was as follows: the tube shell with the gain chip was positioned on the fixture, and then the coupling was monitored through a polarization-maintaining fiber. The laser power was measured through the fiber. The multi-dimensional moving nozzle precisely positioned the collimating lens to maximize the laser power, and then the lens was fixed by the dispensing and curing of UV glue. This method was equally applicable to the passive devices such as the F–P, IF, and mirror, ensuring high performance and stability throughout the optical system. Figure 3a shows the process of installing a collimating lens by the automatic coupling device, while Figure 3b is a partial enlargement of the stage, Figure 3c shows the internal structure of the IF–ECDL manufactured by this automatic coupling device, and Figure 3d shows an overview of the IF–ECDL.

### 3.2. Experimental Results and Analysis

**Figure 4:**
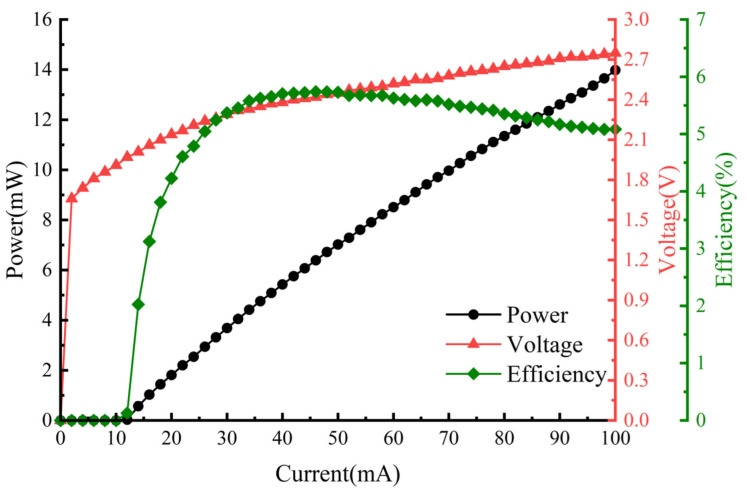
PIV Curve of IF–ECDL.

Both the 2510-AT Thermoelectric Cooler (TEC) SourceMeter from LAILE Photoelectric Co., Ltd. (Wuhan, China) and the B2901A SourceMeter from Keysight (Santa Rosa, CA, USA) were used for regulating the temperature and supplying the driving current during the experiment. The TEC temperature control was set at 25 °C, and the driving current was increased from 0 to 100 mA with a scan step of 2 mA. The current-power-voltage relationship (PIV curve) and electro-optic conversion efficiency of the IF–ECDL were measured at room temperature, as shown in Figure 4. The threshold current of this laser was 12 mA. Upon reaching the threshold current, the output power of the IF–ECDL increased linearly as the driving current was increased. For a driving current of 100 mA, the output power reached 14 mW with a slope efficiency of 0.14 mW/mA. However, higher driving currents, while potentially increasing output power further, posed a risk of device damage. Indeed, the laser was damaged after briefly reaching an output power of 27.8 mW at a driving current of 200 mA during experimentation. When the driving current exceeded the threshold current, the optical-to-electrical conversion efficiency increased rapidly as the current increased, reaching a maximum of 5.74% at 40 mA, and then remained stable with a slight decrease thereafter.

**Figure 5:**
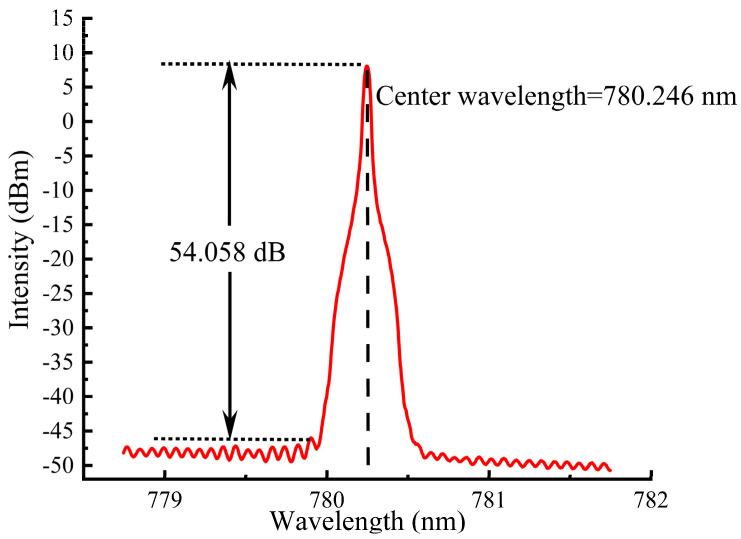
Spectra of IF–ECDL at a driving current of 60 mA and TEC temperature of 22.5 °C.

The AQ6370D optical spectrum analyzer from Yokogawa was employed to assess the spectral characteristics of the IF–ECDL operating in continuous wave mode at a driving current of 60 mA and with a temperature controlled by a TEC set to 22.5 °C. The results of the stimulated emission spectrum were plotted in Figure 5. It can be observed that the laser has a single-mode output with a center wavelength precisely measured at 780.246 nm, corresponding to the D2 transition line of rubidium (Rb) atoms. The SMSR was as high as 54.058 dB, which indicates the extremely high spectral purity of the laser.

**Figure 6:**
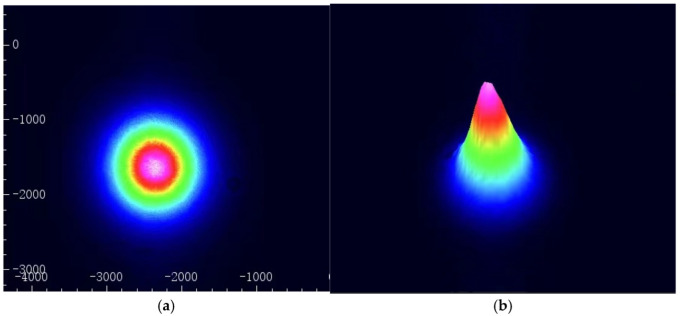
Energy distribution of IF–ECDL. (**a**) Two-dimensional energy distribution; (**b**) Three-dimensional energy distribution.

A Thorlabs BC106N-VIS/M camera beam profiler was employed to assess the output energy distribution of the IF–ECDL fiber tip at a temperature of 22.5 °C and a drive current of 60 mA. The results are shown in Figure 6, where Figure 6a shows the output beam profile of the IF–ECDL after passing through the pigtail, and Figure 6b shows the three-dimensional contour distribution of the laser output beam’s energy. The result indicates that our manufacturing IF–ECDL has a fundamental mode output through the pigtail and has high beam quality.

**Figure 7:**
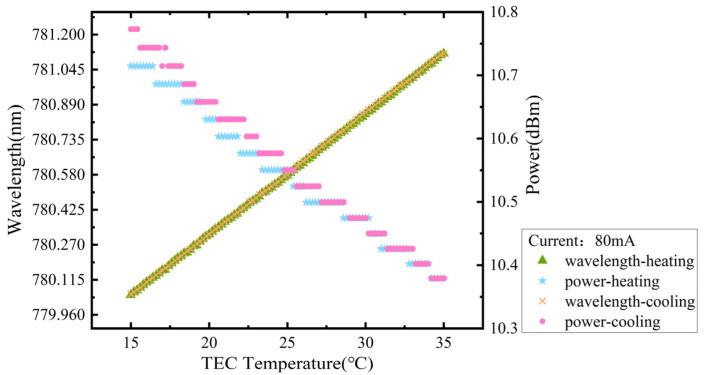
Curves of the bidirectional temperature scanning of the IF–ECDL.

Both the Keithley B2901A SourceMeter and the T2510-AT TEC SourceMeter were employed to conduct bidirectional temperature scans of the IF–ECDL, with a working current set at 80 mA. The temperature of the scan range for the TEC was set from 10 °C to 35 °C. The results are shown in Figure 7. Throughout both the heating and the cooling processes, the wavelength changed linearly without any mode hopping. This indicates a stable output from the IF–ECDL. By analyzing the heating and cooling scans, a continuously tunable wavelength range of up to 1.07 nm (527 GHz) was calculated, with wavelength-temperature changing rates of 53.4 pm/°C and 53.2 pm/°C, respectively, during the heating and cooling processes. During the heating process, a slight decrease in output power was observed. This phenomenon can be attributed primarily to the increase in diffusion energy of the injected carriers as the temperature rises. This, in turn, leads to a decrease in both the gain and laser efficiency. Additionally, power measurements exhibited a staircase-like pattern throughout the testing period. The reason for that is that the pre-controlled TEC temperatures reached the specified set points in the increment of 0.2 °C, but the power readings were taken after thermal stabilization at each set point.

**Figure 8:**
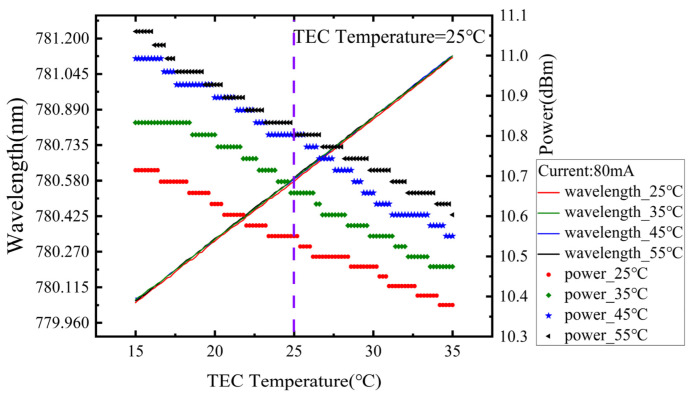
Temperature rising scanning curves at different ambient temperatures.

By positioning the IF–ECDL on a heating platform, the IF–ECDL’s temperature increase scan under different ambient temperatures was conducted. The results are shown in Figure 8. After an increase in ambient temperature was observed, the output wavelength of the IF–ECDL showed a slight upward trend, with a temperature drift coefficient of 0.53 pm/°C. This phenomenon arises due to the fact that the TEC regulates temperature solely at a designated point within the resonant cavity, whereas fluctuations in ambient temperature result in alterations to the temperature field distribution throughout the entire resonant cavity. This variation of the temperature field further prompts the laser’s output wavelength to shift towards longer. Changes in wavelengths and output power indicate that its overall operating mode shifts with ambient temperature variations. It is noteworthy that the TEC temperature scan range is set to a limited extent to ensure the safety of the laser components, preventing direct observation of the complete evolution of the entire operating mode under extreme conditions. Therefore, effective heat dissipation measures and temperature compensation strategies need to be implemented to improve the temperature control accuracy of the TEC in practical applications, thereby stabilizing the output wavelength and power of the laser and ensuring its reliable operation under different environmental conditions.

**Figure 9:**
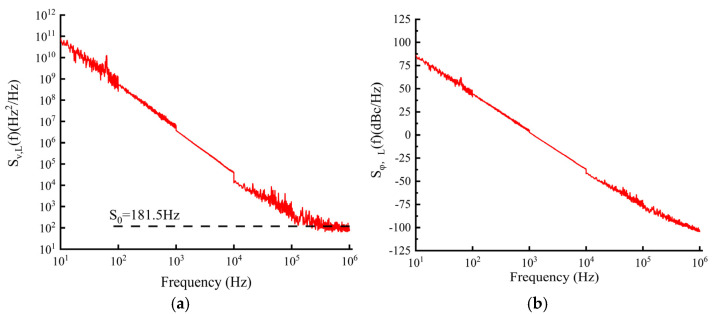
Test results of frequency noise and phase noise of IF–ECDL. (**a**) Test results of frequency noise; (**b**) Test results of phase noise.

In the experiment, the OE4000 test equipment from OEwaves, Inc. (Pasadena, CA, USA) was employed to perform frequency noise and phase noise assessments on the IF–ECDL. The test results are shown in Figure 9, which allowed for further calculation of the linewidth characteristics of the IF–ECDL. The observed frequency noise power spectral density $S_{v,L}\left(f\right)$ was plotted as shown in Figure 9a and showed a decrease in frequency from 10 Hz to 1 MHz, corresponding to white noise. We can deduce the fast linewidth (Δv) of the laser using the following equation [15]:

(8) $$\int_{\Delta v/2}^{\infty }S_{\phi ,L}\left(f\right)df=\frac{2}{\pi }$$

where $S_{\phi ,L}\left(f\right)=S_{v,L}\left(f\right)/f^{2}$ is the IF–ECDL phase noise. The low-frequency noise of the laser is aroused by external disturbance. The level of white noise corresponds to the natural linewidth of the laser. The linewidth of the IF–ECDL was calculated by the following equation [25,29]:

(9) $$\Delta v=\pi S_{0}$$

where $S_{0}=181.5\;\mathrm{HZ}$ is the white noise level and is obtained from Figure 9a. The linewidth of the IF–ECDL was calculated to be 570 Hz. The test results for the phase noise power spectral density of the IF–ECDL are presented in Figure 9b, further substantiating the correlation between phase noise and frequency noise as indicated by the experimental data.

## 4. Conclusions

A compact, high-performance 780 nm narrow linewidth IF–ECDL featuring a butterfly package structure were successfully achieved by employing an F–P in conjunction with an IF as frequency selection elements. Experimental results demonstrate that the IF–ECDL can achieve continuous wavelength tuning within a certain range through both temperature and current control. It exhibits excellent single-mode characteristics across the entire current and temperature tuning range. At a limited drive current, the output power can reach 14 mW, with an SMSR of 54 dB and a temperature tuning range of 527 GHz (1.068 nm). In thiexperiment, we achieved a narrowest laser output linewidth of 570 Hz, which is better than the results reported in current articles for other IF–ECDLs at 780 nm.

The IF–ECDL demonstrates exceptional performance in terms of its dimensions, mass, output power, single-mode stability, frequency purity, and tuning range, thereby offering an ideal solution for the applications of coherent optical communication and quantum precision measurement.

## Data Availability

Data are contained within the article.
